# Exploring the Perception of Continuity of Care in Midwifery in Abu Dhabi, United Arab Emirates

**DOI:** 10.7759/cureus.93055

**Published:** 2025-09-23

**Authors:** Fatima Elmi, Anila Aravindan, Saima Qayoom, Anupama Bondili

**Affiliations:** 1 Obstetrics and Gynaecology, Tawam Hospital, Abu Dhabi, ARE

**Keywords:** community midwifery, continuity of care, healthcare delivery, hospital readmissions, maternal health, midwifery, mixed-methods research, patient-centered care, postpartum care, united arab emirates

## Abstract

Background

Continuity of care (COC) in midwifery refers to a model in which a known midwife provides ongoing support to a woman throughout her pregnancy, labor, and postpartum period. This approach is believed to enhance the overall quality of care and support positive experiences for both mothers and newborns. However, its implementation varies globally, and it is not widely adopted in some regions, including the United Arab Emirates (UAE). This study explored the feasibility and perception of midwifery-led COC in Abu Dhabi and assessed its potential to reduce hospital readmissions.

Methodology

A concurrent, mixed-methods, triangulation design was employed. Qualitative data were gathered via semi-structured interviews and focus groups with 40 maternity care professionals. Quantitative data included surveys from 110 women and a retrospective review of 508 maternal and neonatal records. Thematic analysis was conducted using NVivo; quantitative data were analyzed with SPSS version 27 (IBM Corp., Armonk, NY, USA).

Results

Healthcare professionals expressed strong support for midwifery-led COC, with 97.5% advocating its implementation and over 75% of midwives confident in delivering community-based care. Women also favored continuity models, with 90.9% expressing a preference for ongoing care from a known midwife. Readmission data showed common causes such as jaundice, sepsis, and wound infections, many of which are preventable with early postnatal intervention. Readmission rates increased during the COVID-19 pandemic, highlighting gaps in follow-up care.

Conclusions

Midwifery-led COC is both feasible and well-received in the UAE context. Implementing community-based midwifery services could enhance maternal and newborn outcomes, reduce preventable readmissions, and increase patient satisfaction. Policymakers should prioritize regulatory reform, workforce development, and digital innovation to support COC implementation.

## Introduction

Continuity of care (COC) in midwifery refers to a model where a known midwife provides care throughout the antenatal, intrapartum, and postnatal periods. This approach fosters trust, reduces care fragmentation, and leads to improved outcomes for women and newborns. Globally, midwifery-led COC has been associated with lower rates of preterm birth and medical interventions, alongside increased patient satisfaction, maternal confidence, and breastfeeding success [1-3].

The World Health Organization (WHO) advocates for midwife-led continuity models as a highly effective strategy to improve maternal and neonatal outcomes, particularly in resource-constrained settings [4]. A 2016 Cochrane review found that women under midwifery-led care were less likely to undergo episiotomy, instrumental birth, or regional analgesia and more likely to experience spontaneous vaginal delivery and continuity with a known provider [1]. These models have also been shown to enhance maternal autonomy and reduce healthcare costs [5,6].

Despite this strong evidence, the Gulf region, including the United Arab Emirates (UAE), has seen limited implementation of midwifery COC. Maternity services in the UAE are predominantly obstetrician-led and hospital-based, with minimal support for community midwifery roles [7]. A national assessment reported systemic barriers to midwifery care, including regulatory gaps, training limitations, and cultural preferences for physician-led care [8]. Tawam Hospital in Al Ain, a tertiary facility managing over 3,000 births annually, represents a significant portion of maternity care delivery in Abu Dhabi.

Frequent postpartum readmissions for both mothers and newborns, often for preventable conditions such as jaundice, feeding difficulties, and wound infections, point to deficiencies in postnatal follow-up [9,10]. The Royal College of Midwives underscores the value of early community-based visits in preventing such outcomes [11]. The COVID-19 pandemic further strained maternity services, revealing the need for adaptable, home-based care options [12].

This study aims to explore the perceptions of healthcare professionals and women toward midwifery-led COC in Abu Dhabi and assess hospital readmissions in relation to the absence of community-based midwifery services.

## Materials and methods

Study design

A concurrent, triangulation, mixed-methods design was adopted to explore the feasibility and perception of implementing midwifery-led COC in Abu Dhabi, UAE. This approach enabled the collection and analysis of both qualitative and quantitative data in parallel, enhancing the depth, validity, and reliability of findings through methodological triangulation.

Study setting

This study was conducted at Tawam Hospital, a tertiary referral center and the only public maternity facility in Al Ain. Participants were drawn from various hospital departments providing maternity care, including outpatient clinics, inpatient wards, and the neonatal unit. Laboring women were excluded as per institutional guidelines, classifying them as a vulnerable group.

Study population and sampling

A total of 150 participants were recruited, including 40 healthcare professionals and 110 women receiving maternity care. The professionals represented key stakeholders in maternity services, such as midwives, obstetricians, nurse managers, lactation consultants, and senior administrators. Women were selected from antenatal, postnatal, and lactation clinics, as well as maternity and neonatal wards.

A non-probability sampling approach was used, combining purposive, heterogeneous, and convenience sampling. Healthcare professionals were purposefully selected to capture diverse expertise, while focus groups were organized homogeneously by role to foster open discussion. Patients were recruited based on accessibility during routine care visits. Additionally, 508 maternal and neonatal medical records were retrospectively reviewed to assess readmission trends.

Sample size and sampling strategy

A mixed sampling approach was used, reflecting the mixed-methods design. For the qualitative component, 40 healthcare professionals were purposively sampled to ensure representation of frontline midwives, nurse managers, physicians, lactation consultants, and senior leaders; qualitative sample size was determined by the aim to reach thematic saturation across role-homogeneous focus groups and key-informant interviews. The women’s survey (n = 110) was a convenience sample of clinic attendees during the study period; all eligible women who consented were included. Although no formal a priori hypothesis-driven power calculation was performed, the achieved sample provided sufficient precision to estimate proportions with a margin of error of approximately 9% at the 95% confidence level. The retrospective chart review (n = 508) included all maternal and neonatal records meeting the inclusion criteria during April 2019-March 2021 and represents the available population for readmission analysis.

Data collection

Primary Data

Between March and September 2021, qualitative data were collected through eight focus groups and four individual interviews with healthcare professionals, conducted via Microsoft Teams due to COVID-19 restrictions. Interview guides covered key themes such as perceptions of COC, readiness for implementation, and potential barriers. Demographic data were captured via self-administered questionnaires using Qualtrics, which also facilitated secure electronic consent.

Patient data were collected using a structured 15-item survey developed and distributed via QR code through Qualtrics. Survey items assessed demographic background, perceptions of midwifery-led care, and attitudes toward COC. Five antenatal and five postnatal women were interviewed during the pilot phase to refine the questionnaire. All 110 patients invited to participate completed the survey.

Secondary Data

A retrospective chart review of 508 medical records was performed for deliveries occurring between April 2019 and March 2021. Data were extracted from the hospital’s electronic medical record system and included maternal and neonatal readmissions within 28 days of discharge. This review aimed to identify patterns and causes of readmission and assess potential gaps that could be addressed through COC models, including the comparison of pre- and intra-pandemic readmission rates.

Data analysis

Qualitative data were analyzed thematically using NVivo software. Transcripts were coded inductively, and themes were refined through iterative analysis to ensure alignment with research objectives. For the qualitative data, transcripts were read repeatedly and coded inductively using NVivo software. Initial codes were generated line by line, then compared across transcripts to identify similarities and differences. Codes were subsequently grouped into broader categories, which were refined into themes and sub-themes through iterative discussions among the research team. Discrepancies in coding were resolved by consensus, and thematic saturation was considered reached when no new codes or concepts emerged from the data.

Quantitative data were analyzed using SPSS Statistics version 27 (IBM Corp., Armonk, NY, USA). Descriptive statistics summarized participant characteristics, and inferential tests, including chi-square and independent sample t-tests, were used to explore associations. A p-value <0.05 was considered statistically significant.

Research strategies and philosophical approach

Grounded Theory was employed to generate a conceptual framework based on the views of healthcare professionals and patients. An Archival Research strategy supported the analysis of structured hospital data. The study followed an abductive approach, integrating inductive insights from qualitative data with deductive reasoning informed by existing theory and clinical experience.

Ethical considerations

Ethical approval was obtained from the Department of Health, Abu Dhabi (reference number: DOH/CVDC/2021/931). Written informed consent was obtained from all participants. Confidentiality was maintained through anonymized data handling, and COVID-19 safety protocols were observed during all phases of data collection.

## Results

Participant characteristics

A total of 40 healthcare professionals participated, with midwives comprising the majority (n = 28, 70%), including staff midwives, charge midwives, midwife educators, and nurse managers. The remainder were physicians (n = 6, 15%), lactation nurses (n = 4, 10%), and senior healthcare leaders (n = 2, 5%). Most participants (n = 26, 65%) had 16-35 years of clinical experience, and 17.5% (n = 7) had over 36 years of experience. Only 40% (n = 16) reported prior community midwifery experience, primarily gained in Nigeria, South Africa, and the United Kingdom, averaging two to three years of practice. Among midwives providing direct patient care (n = 27), 78% expressed confidence delivering community midwifery services within the UAE context. Support for implementing community-oriented midwifery care (COC) models was high (97.5%) (Table 1).

**Table 1:** Characteristics of healthcare professionals.

Characteristics	Frequency	Percentage
Profession/Position		
Staff Midwife	7	17.5%
Charge Midwife	18	45.0%
Nurse Manager	2	5.0%
Midwife Educator	1	2.5%
Specialist Physician	1	2.5%
Consultant Physician	5	12.5%
Assistant Director of Nursing	1	2.5%
Chief Nursing Officer	1	2.5%
Lactation Nurse	4	10.0%
Experience as a healthcare professional		
6–15 years	7	17.5%
16–25 years	15	37.5%
26–35 years	11	27.5%
>36 years	7	17.5%
Community midwifery experience		
Yes	16	40.0%
No	24	60.0%
Country of Community midwifery experience		
Nigeria	6	37.5%
South Africa	3	18.8%
Jordan	1	6.3%
Palestine	1	6.3%
United Kingdom	4	25.0%
Fiji	1	6.3%
Community midwifery experience		
<1 year	3	18.8%
2–3 years	9	56.3%
4–10 years	1	6.3%
11–15 years	3	18.8%
Community midwifery should be implemented in the UAE		
Yes	39	97.5%
No	1	2.5%
Confident to be a community midwife in the UAE? (For midwives only)		
Yes	21	52.5%
Not applicable	13	32.5%
No	6	15.0%

Thematic analysis

**Figure 1:**
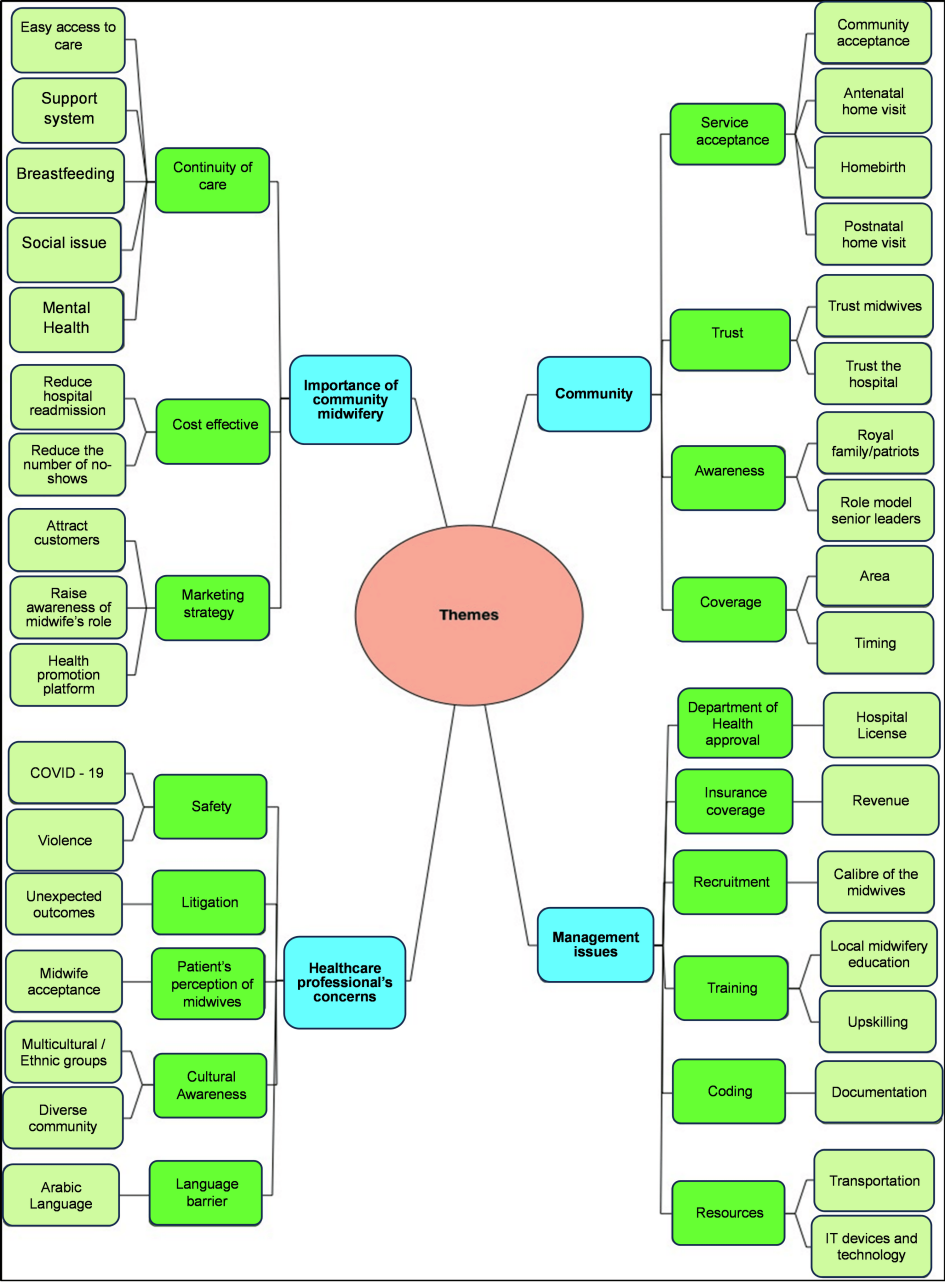
Themes from healthcare professionals’ interviews.

A total of 12 semi-structured interviews were conducted. Qualitative analysis using NVivo identified the following four main themes: (1) perceptions of community midwifery; (2) barriers to implementation; (3) education and training needs; and (4) support systems and interprofessional collaboration (Figure 1).

Theme 1: Importance of Community Midwifery

Participants emphasized COC as a key benefit, enabling early detection and management of clinical and psychosocial complications during pregnancy and postpartum. Gaps in follow-up for high-risk patients were noted, partly due to hospital policies limiting contact with non-attendees. Early postnatal discharge hindered the timely detection of postpartum depression. Community midwives were considered vital for early screening, referral, and breastfeeding support. Despite high in-hospital breastfeeding initiation (>80%), lack of post-discharge follow-up led to complications such as neonatal dehydration and jaundice. Participants suggested COC could enhance institutional reputation if endorsed by health authorities.

Theme 2: Community Challenges

Barriers included low public awareness and physician-centered care dominance. Postnatal home visits were culturally accepted, while antenatal care and homebirths faced skepticism due to family living arrangements, privacy concerns, and stigma associating homebirth with low socioeconomic status. A phased approach, starting with postnatal visits and expanding antenatal care, was recommended.

Theme 3: Management and Logistical Concerns

Regulatory approval and financial sustainability were major issues. Participants questioned licensing by the Department of Health (DOH) and insurance coverage for home-based care. Capacity building was needed, as most UAE midwives lacked formal community care training. Suggested strategies included recruitment, retraining, equipment acquisition, and cost-effectiveness evaluation.

Theme 4: Safety and Professional Concerns

Safety during home visits, especially during COVID-19, was a concern due to unscreened environments and a lack of protective measures. Legal liability in adverse events was unclear, causing hesitancy. Cultural perceptions of midwifery as subordinate to physicians affected trust in midwife-led home care. Recruiting Arabic-speaking midwives was emphasized for culturally sensitive communication.

Secondary data analysis

Among 508 maternal and neonatal records analyzed for readmissions within 28 days post-discharge, delivery mode significantly influenced readmission rates (p < 0.05). Readmissions increased during the COVID-19 pandemic, indicating disruption of postpartum care. Maternal readmissions were mainly for sepsis and wound infections; neonatal readmissions were primarily due to jaundice and feeding issues. These findings support the need for community midwifery to improve postpartum outcomes.

Participant demographics (survey)

Among 110 surveyed women, 60% (n = 66) were pregnant and 40% (n = 44) were postnatal. Most were aged 18-39 years (81%), predominantly Emirati (79%), insured under Thiqa, and over half were unemployed. Overall, 41% had a bachelor’s degree or higher (Table 2).

**Table 2:** General characteristics of the study participants (N = 110).

	Frequency	Percentage
Age		
18–29	38	35%
30–39	51	46%
40–45	19	17%
>46	2	2%
Nationality		
Emirati	87	79%
Non-Emirati	23	21%
Health insurance		
Thiqa	95	86%
Daman	12	11%
Self-pay	0	0%
Others	3	3%
Number of children		
None	19	17%
1–2	42	38%
3–5	26	24%
>5	23	21%
Level of education		
Less than high school	8	7%
High school certificate	31	28%
Higher diploma	19	17%
Bachelors (BSc, BEd, etc.)	45	41%
Masters (MA, MSc, etc.)	7	6%
Doctorate (PhD)	0	0%
Employed		
Yes	46	42%
No	64	58%

Knowledge and perceptions of midwifery

**Figure 2:**
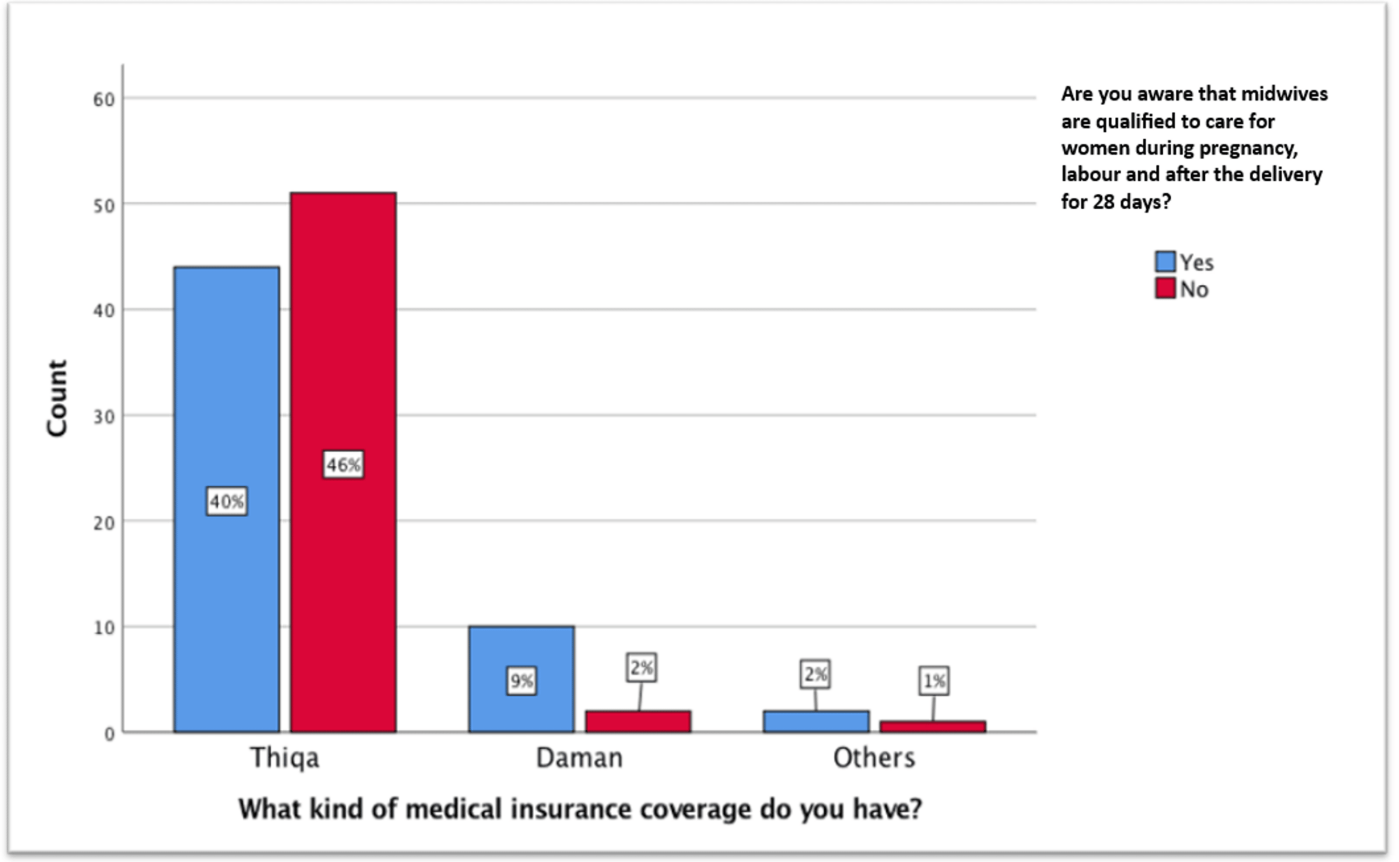
Insurance versus scope of the midwife.

About 69.1% were aware of midwives’ roles, but only 50.9% recognized their antenatal and postnatal care roles. Physician preference for delivery was reported by 59.1%, reflecting limited perceived midwifery roles. Higher education correlated with greater midwifery knowledge (χ² = 9.57, df = 4, p = 0.048). Insurance type influenced awareness; Thiqa holders were less informed than Daman or others (χ² = 6.15, df = 2, p = 0.046) (Figure 2).

Delivery provider preferences and parity

No significant association was found between parity and delivery provider preference (p > 0.05). Primigravida women preferred physicians (73.7%), while multiparous women (≥5 children) preferred midwives (56.5%) (Table 3).

**Table 3:** Cross-tabulation of the number of children versus preference for a doctor or midwife.

Would you prefer a midwife or a doctor to deliver your baby?			Midwife	Doctor	Total
How many children do you have?	None	Count	5	14	19
		% within how many children do you have?	26.3%	73.7%	100%
	1–2	Count	17	25	42
		% within how many children do you have?	40.5%	59.5%	100%
	3–4	Count	10	16	26
		% within how many children do you have?	38.5%	61.5%	100.%
	>5	Count	13	10	23
		% within how many children do you have?	56.5%	43.5%	100%
Total		Count	45	65	110
		% within how many children do you have?	40.9%	59.1%	100%

Continuity of care and home visits

Support for COC was high (90.9%), with 82.7% anticipating family and community acceptance. Preferences for home visits were 70% antenatal and 90% postnatal. However, 67.3% opposed homebirth due to safety and cultural concerns (Tables 4, 5).

**Table 4:** Relationship between women’s characteristics and their perception of continuity of care.

Women’s perception of continuity of care	Frequency	Percentage
Prefer continuity of care		
Yes	100	90.9%
No	10	9.1%
Antenatal follow-up		
Home visit	67	60.9%
Hospital visit	43	39.1%
Postnatal follow-up		
Home visit	71	64.5%
Hospital visit	39	35.5%
Home birth		
Yes	14	12.7%
No	74	67.3%
Maybe	22	20.0%
Continuity of care acceptance by family/community		
Yes	91	82.7%
No	19	17.3%

**Table 5:** Chi-square to test the relationship between the women’s characteristics and their perception of continuity of care.

	Continuity of care acceptance	Antenatal follow-up	Postnatal follow-up	Home birth	Continuity of care acceptance by family/community
Age	0.65	0.063	0.085	0.911	0.575
Nationality	1	1	0.866	0.304	0.769
Health insurance	0.553	0.415	0.257	0.737	0.476
Number of children	0.23	0.441	0.89	0.789	0.409
Level of education	0.36	0.544	0.693	0.756	0.443
Employment	0.071	0.168	0.082	0.055	0.211
N ***p < 0.001 , **p < 0.01 , *p < 0.05	110	110	110	110	110

Hospital readmissions

Maternal Readmissions

**Figure 3:**
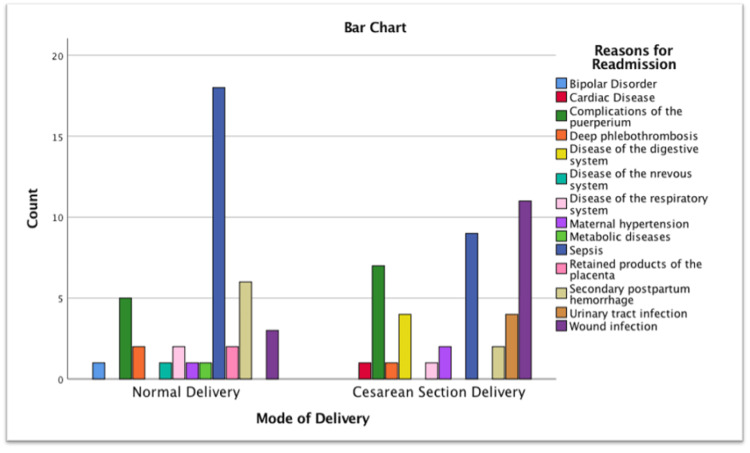
Mode of delivery versus reasons for readmission.

A total of 84 postpartum readmissions occurred over the two-year study period. During COVID-19, the proportion of normal vaginal deliveries decreased from 71.6% pre-COVID-19 to 64.7% during COVID-19, while cesarean deliveries increased from 28.4% to 35.3%, despite a modest 1.8% rise in overall delivery numbers. Maternal readmission rates declined from 1.64% before COVID-19 to 1.10% during COVID-19, although this difference was not statistically significant (χ² = 0.44, df = 1, p = 0.50) (Table 6). Causes of readmission differed significantly by mode of delivery (χ² = 24.91, df = 13, p = 0.024), with sepsis being the most common following vaginal delivery (42.9%) and wound infections most frequent after cesarean section (26.2%) (Figure 3).

**Table 6:** Number of readmissions.

	Before COVID-19 (April 2019 to March 2020)	During COVID-19 (April 2020 to March 2021)	Total
Total delivery (women )	3,042	3,098	6,140
Total birth (newborns )	3,184	3,219	6,403
Normal delivery	2,178	2,005	4,183
Cesarean delivery	864	1,093	1,957
Total women readmitted	50	34	84
Readmitted for normal delivery	27	15	42
Readmitted for cesarean section delivery	23	19	42
Readmitted newborn	200	224	424
Rate of readmission for women	1.64%	1.10%	1.37%
Rate of readmission for normal delivery	1.24%	0.75%	1.00%
Rate of readmission for cesarean delivery	2.66%	1.74%	2.15%
Rate of readmission for newborns	6.28%	6.96%	6.62%

Newborn Readmissions

Among 424 neonatal readmissions, jaundice was the leading cause, representing the majority of cases. During COVID-19, the proportion of readmissions due to dehydration, acute respiratory failure, and ABO isoimmunization increased significantly (χ² = 55.55, df = 10, p < 0.05). Overall, neonatal readmission rates rose slightly from 6.28% pre-COVID-19 to 6.96% during COVID-19 (Table 6). Reasons for readmission varied significantly by the initial discharge unit (χ² = 83.83, df = 30, p < 0.05), with most neonates readmitted after discharge from the obstetrics unit. The average time to readmission was shorter during the pandemic, decreasing from 8.72 days pre-COVID-19 to 6.24 days during COVID (p = 0.001). Although male neonates were more frequently readmitted with jaundice, this association was not statistically significant (p = 0.438).

Impact of COVID-19 on health insurance and readmissions

**Figure 4:**
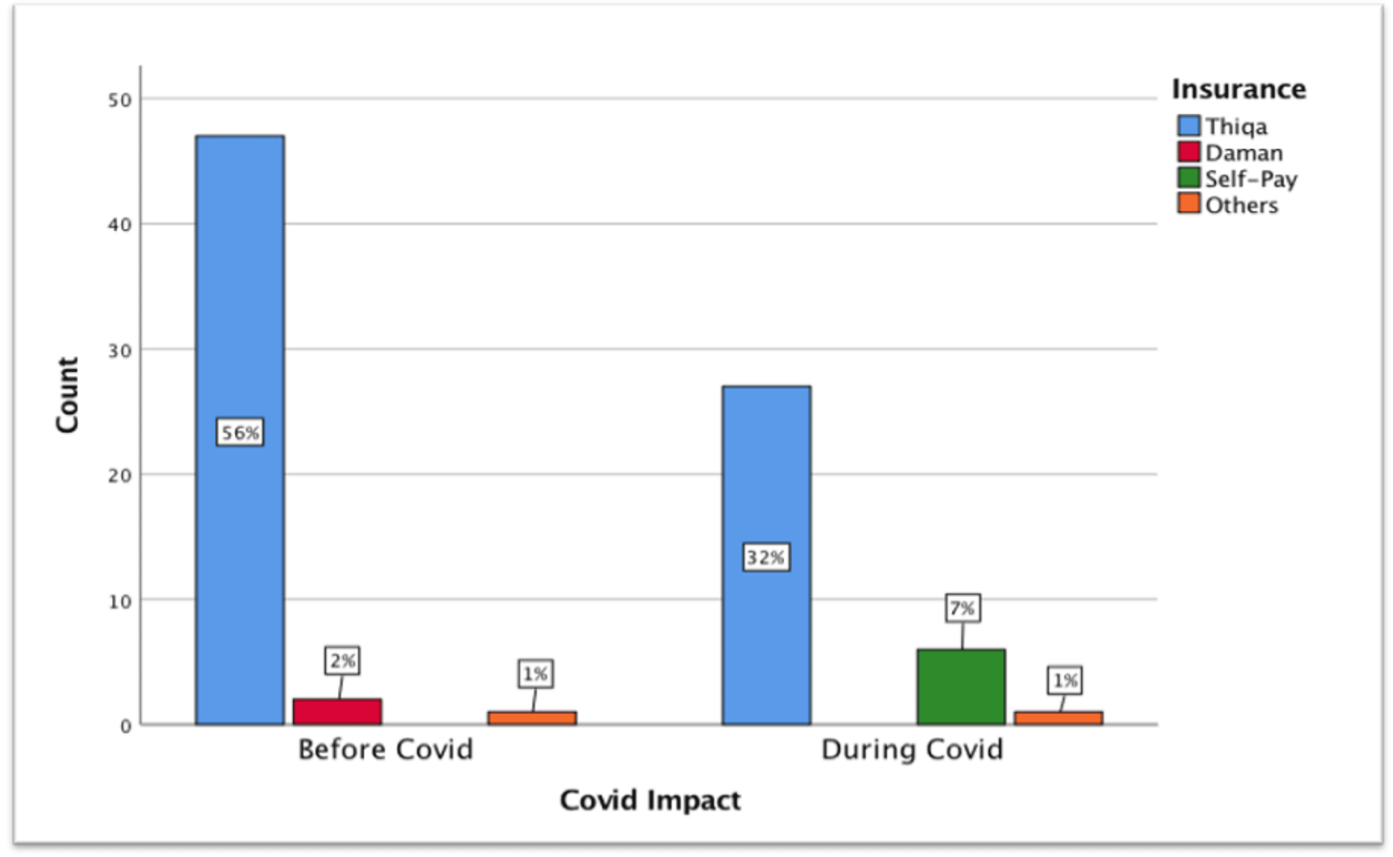
Impact of COVID-19 on health insurance.

During the pandemic, readmissions among Thiqa-insured women decreased by 24%, Daman by 2%, while self-pay readmissions increased by 7% (χ² = 10.75, df = 3, p = 0.013) (Figure 4).

Readmission timing by delivery mode

**Figure 5:**
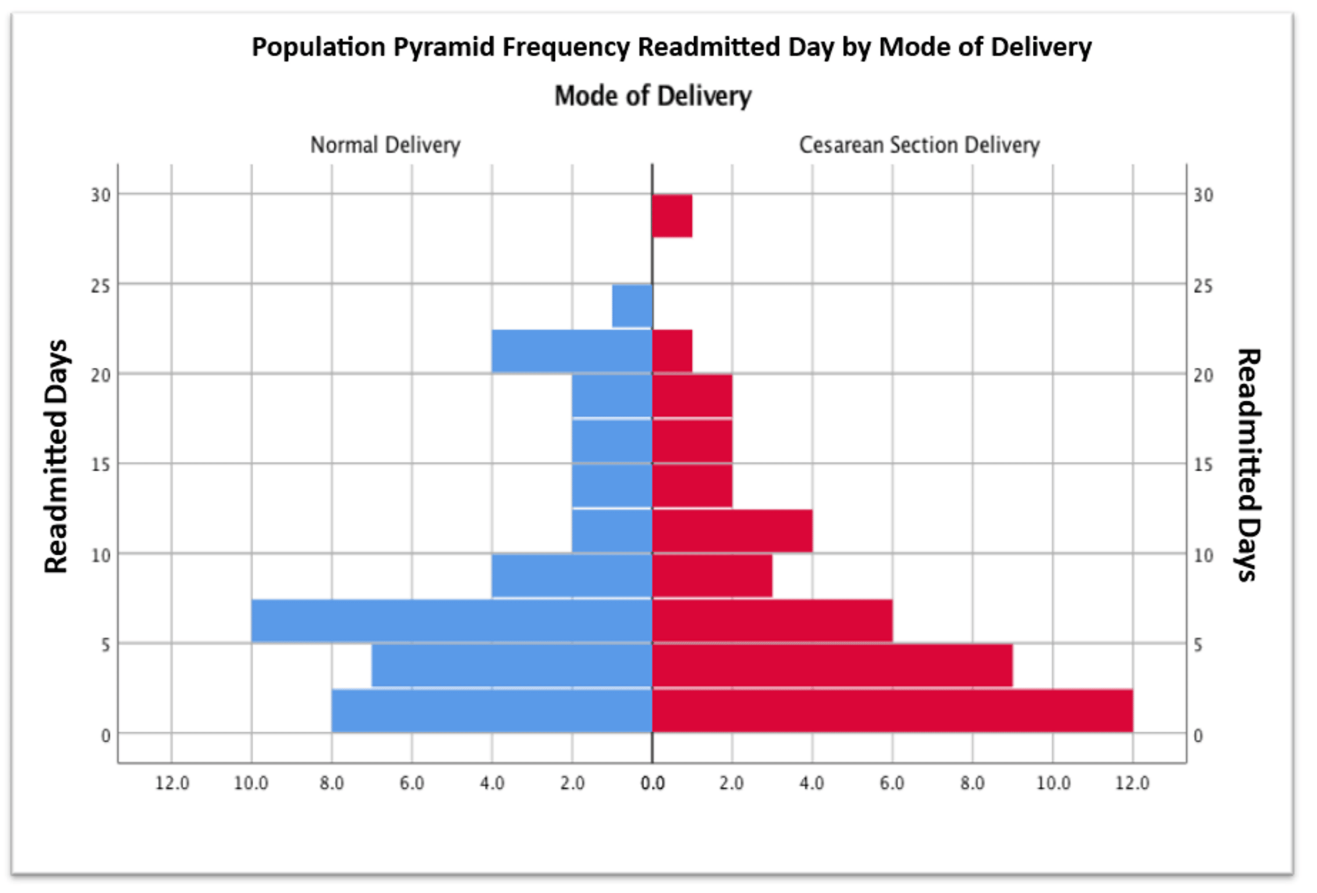
Population pyramid for mode of delivery and readmitted days.

Mean time to readmission was slightly shorter after cesarean section (7.53 days) than after vaginal delivery (8.61 days), though not significant (t = 0.73, df = 82, p = 0.466). Most readmissions occurred within six to seven days postpartum (Figure 5).

Newborn readmissions

Among 424 newborn readmissions, jaundice was the most frequent cause. Significant increases in dehydration, acute respiratory failure, and ABO isoimmunization were noted during COVID-19 (χ² = 55.55, df = 10, p < 0.05). Readmission reasons varied significantly by initial discharge unit (χ² = 83.83, df = 30, p < 0.05), with most neonates admitted from the obstetrics unit. Neonates were readmitted sooner during the pandemic (6.24 days vs. 8.72 days pre-pandemic, p = 0.001). Though males had more jaundice readmissions, no significant gender association was found (p = 0.438).

## Discussion

This study demonstrates strong support for midwifery-led COC in Abu Dhabi and highlights its feasibility within the UAE context. The triangulated mixed-methods design, incorporating perspectives from healthcare professionals, women, and hospital readmission data, strengthens the reliability of findings. Both groups favored COC, citing its role in reducing postnatal complications, improving maternal and newborn outcomes, and enhancing satisfaction. A majority of women expressed willingness to receive community-based care and preferred a known midwife throughout their maternity journey. While the themes were presented individually, they can also be understood as barriers (e.g., regulatory gaps, logistical and safety concerns) and facilitators (e.g., women’s preference, professional support, and feasibility of postnatal visits), which collectively influence the feasibility of implementing continuity of care in the UAE.

The study’s results are consistent with international evidence. Globally, midwifery-led continuity models have demonstrated reductions in preterm births, neonatal mortality, and medical interventions [1,2]. A Cochrane review confirmed that women under midwifery-led care were more likely to experience spontaneous vaginal births and report higher satisfaction [1]. In countries such as Australia and New Zealand, these models have been associated with improved breastfeeding initiation, reduced cesarean rates, and shorter hospital stays [3,4]. These benefits are similarly observed in low- and middle-income countries, where such care models enhance maternal outcomes and equity in healthcare access [5].

Despite strong global evidence, maternity care in the UAE remains predominantly obstetrician-led, with community-based midwifery services largely absent. This study found that healthcare professionals recognize the need for structural reform, with many expressing interest in participating in new models of care. These findings echo those of Brownie et al., who identified gaps in midwifery regulation and training in the UAE [7].

To support the implementation of community-based care, it is essential to develop licensing frameworks, standardized training, and public education initiatives. Lessons can be drawn from the United Kingdom’s National Health Service, which has demonstrated the cost-effectiveness and safety of midwife-led care models [13,14].

Digital health technologies present additional opportunities to strengthen continuity of care. Tools such as telehealth and mobile applications can facilitate remote postnatal follow-up, virtual consultations, and early detection of complications, especially in underserved areas [15-17]. Integrating digital tools into community midwifery services can enhance access and patient engagement while reducing the burden on hospital systems [18].

This study is limited to a single tertiary hospital, and the participant sample, though diverse, was relatively small and primarily Emirati. These factors may limit the generalizability of the findings to other regions within the UAE. Additionally, further economic analysis is warranted to assess the cost-effectiveness and resource implications of community-based midwifery services [19].

Further implementation research is needed to evaluate pilot models of community midwifery in the UAE, including outcome monitoring, service utilization, and patient-reported experiences. Training programs should be expanded to prepare midwives for home-based care roles, and collaboration with international midwifery organizations can help adapt best practices. These efforts will support the development of a sustainable, high-quality maternal care system that aligns with global standards and meets local needs.

## Conclusions

This study provides evidence that midwifery-led COC is perceived as feasible in the Abu Dhabi context, based on the positive attitudes and readiness of healthcare professionals to support community-based midwifery and the strong preference of women for continuous, personalized care. While hospital and newborn readmission data do not directly determine feasibility, they highlight deficiencies in current postnatal care, particularly regarding breastfeeding support and neonatal jaundice, that community midwifery could potentially address. We do not infer that these outcomes would have been prevented by home visits, but the readmission data help contextualize areas where COC may improve maternal and neonatal support. Implementation of COC models in the UAE holds promise for improving maternal and neonatal outcomes, reducing hospital readmissions, and enhancing patient satisfaction. These findings align with international evidence supporting midwifery-led care and suggest that pilot initiatives are warranted. Successful adoption will require regulatory endorsement, targeted workforce training, interprofessional collaboration, and public education. The COVID-19 pandemic emphasized the need for adaptable maternal care services, and community midwifery may offer a sustainable solution. Further research should explore pilot implementation, cost-effectiveness, and integration of digital health technologies to support outreach, follow-up care, and evaluation of outcomes to guide broader implementation.
